# Weakly Supervised Crop Area Segmentation for an Autonomous Combine Harvester

**DOI:** 10.3390/s21144801

**Published:** 2021-07-14

**Authors:** Wan-Soo Kim, Dae-Hyun Lee, Taehyeong Kim, Hyunggun Kim, Taeyong Sim, Yong-Joo Kim

**Affiliations:** 1 Institute of Agricultural Science, Chungnam National University, Daejeon 34134, Korea; wskim0726@gmail.com; 2 Department of Biosystems Machinery Engineering, Chungnam National University, Daejeon 34134, Korea; babina@cnu.ac.kr; 3 Interdisciplinary Program in Cognitive Science, Seoul National University, Seoul 08826, Korea; taehyeong.kim@lge.com; 4 Department of Biomechatronic Engineering, Sungkyunkwan University, Suwon 16419, Korea; 5 Department of Artificial Intelligence, Sejong University, Seoul 05006, Korea; tysim@sejong.ac.kr; 6 Department of Smart Agricultural Systems, Chungnam National University, Daejeon 34134, Korea

**Keywords:** combine harvester, weakly supervised, semantic segmentation, uncut crop edge, path guidance

## Abstract

Machine vision with deep learning is a promising type of automatic visual perception for detecting and segmenting an object effectively; however, the scarcity of labelled datasets in agricultural fields prevents the application of deep learning to agriculture. For this reason, this study proposes weakly supervised crop area segmentation (WSCAS) to identify the uncut crop area efficiently for path guidance. Weakly supervised learning has advantage for training models because it entails less laborious annotation. The proposed method trains the classification model using area-specific images so that the target area can be segmented from the input image based on implicitly learned localization. This way makes the model implementation easy even with a small data scale. The performance of the proposed method was evaluated using recorded video frames that were then compared with previous deep-learning-based segmentation methods. The results showed that the proposed method can be conducted with the lowest inference time and that the crop area can be localized with an intersection over union of approximately 0.94. Additionally, the uncut crop edge could be detected for practical use based on the segmentation results with post-image processing such as with a Canny edge detector and Hough transformation. The proposed method showed the significant ability of using automatic perception in agricultural navigation to infer the crop area with real-time level speed and have localization comparable to existing semantic segmentation methods. It is expected that our method will be used as essential tool for the automatic path guidance system of a combine harvester.

## 1. Introduction

Machine vision is an attractive technology that is widely used to perceive local environments for autonomous navigation in agriculture. It has several advantages compared to GPS-based navigation in that it can provide local guidance in real time without an a prior map of the target area and easily integrate other essential features such as obstacle avoidance [1]. Even if the machine is not fully automated, visual perception can assist the operator by reducing the fatigue that comes with performing tedious and monotonous visual tasks. For example, the combine harvester, which has made a huge contribution to improving agricultural productivity [2], is used for harvesting field crops, and the operator has to constantly observe uncut crop edges to determine the path and control steering [3,4]. Machine-vision-based assistance can guide local path automatically, allowing the operator to focus on other tasks to maximize production and reduce fatigue [5,6].

Research into machine-vision-based assistance or guidance systems for combine harvesters has been going on for the past few decades [1,6]. Methods have been developed based on various data sources: color space [5,7,8] and distance information obtained via stereo camera [1,9,10], LiDAR [11,12,13], and depth camera [14,15]. These usually extract the boundary between the harvested and unharvested areas by detecting the uncut crop area. One of the most promising methods for this is semantic segmentation, which clusters target objects (i.e., area) by conducting pixel-by-pixel classification. Deep learning has vastly improved performance of semantic segmentation by proposing effective model architectures, such as fully convolutional networks (FCNs) [16]. In addition, segmentation models with higher inference speeds and accuracies are proposed every year [17,18,19]. Deep learning has also become a popular approach for automatic visual perception in agriculture due to its robust feature extraction, which can be expected to overcome the limitations of conventional image processing [20]. Several researchers have demonstrated comparable deep-learning model performance in the segmentation of fruit [21], diseases [22], obstacles [23], and navigation paths [24,25]. In particular, Li et al. [14] conducted uncut crop area detection with collision avoidance based on sementic segmentation, thereby showing a practical performance for combine harvesters. They used a network-slimming algorithm based on ICNet to improve segmentation inference [17] in real time.

Deep-learning-based segmentation has proven to be feasible and effective in agriculture, as mentioned above; however, semantic segmentation models usually require a large number of parameters to train the features for pixel-by-pixel classification and large-scale datasets that involve laborious annotation to extract the features for pixel-based predictions [26]. In particular, the front scenes captured during combine harvesting, which are used as datasets to train deep-learning models, have monotonous and similar features; thus, the recorded frames cannot be used as multiple data points due to the similarity between images [24]. In addition, pixel-level annotation for uncut crop areas is not usually easy at the border due to ambiguous edges caused by crop interference, shadows, etc. This border area cannot be explicitly clustered and uncertain annotations can lead to errors in pixel-level segmentation; thus, it is more efficient to train the model to segment the target area based on a partial image-level (group of pixels) supervision rather than pixel-level supervision. For these reasons, we proposed the technique of partial area classification-based segmentation to detect the path area in a citrus orchard [24]. This technique alleviates the problem of data scarcity by replacing the segmentation task, which requires a large dataset in deep learning, with the simple CNN-based task of classifying monotonous patterns in orchard scenes. Although the method achieved a satisfactory performance, it was hard to determine an accurate border edge due to the low spatial resolution of segmentation, which depends on the image patch size (part of the image).

To solve the above-mentioned issues for the path guidance of combine harvesters, we proposed the semantic segmentation of the uncut crop area using weakly supervised learning. The proposed segmentation localized an object implicitly with image-level labeling, which requires less labor than pixel-level segmentation. The target area (i.e., uncut crop area) was inferred from the entire image based on the classification model that was trained using partial images consisting of class-specific areas. The contribution of our approach is that this less laborious segmentation is a suitable deep-learning technique for agricultural navigation, which suffers from the problem of labelled data scarcity. It can also be configured shallowly that is possible to process it practically in real-time.

## 2. Materials and Methods

### 2.1. Task Description

**Figure 1:**
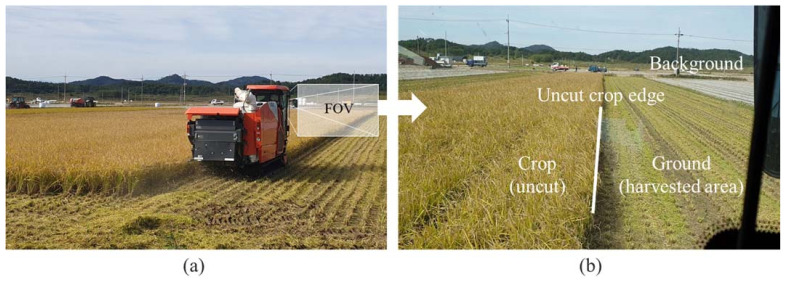
Harvesting scene of a combine harvester in a rice paddy (**a**) and a representative sample image including the operator’s field of view (**b**).

Combine harvesters cut and harvest crops while travelling around the edge of the crop area, as shown in Figure 1a, which shows a combine harvesting scene in a rice paddy. The operator controls the steering based on visual information in field of view (FOV) to track the work path during harvesting. Figure 1b shows major objects in the FOV used as visual clues to determine the path. In general, the combine harvester moves forward by cutting the crop according to the width of the cutter from the uncut crop edge, and the operator adjusts the machine’s longitudinal direction to make it parallel to the uncut crop edge. Thus, machine vision must be able to detect the uncut crop area and its edge in contact with the harvested area for the path guidance system. In this study, the uncut crop area in the FOV (front scene) captured from inside the combine harvester was segmented to determine the uncut crop edge for reference as the work path.

### 2.2. Crop Area Segmentation

Weakly supervised learning can train a model by learning with indirect or weak supervision that can reduce the need for laborious annotation, such as pixel-level labelling for dataset construction in semantic segmentation. One of type of weakly supervised learning used for localization is to train a classification model as used backbone for generating class activation maps (CAMs) for each target category [27]. This not only helps to classify the image into one of the pre-determined classes but also provides weak localization cues. Thus, this method has the advantage of localizing an object in an image with just an image-level label requiring very simple annotation compared to a pixel-level label.

**Figure 2:**
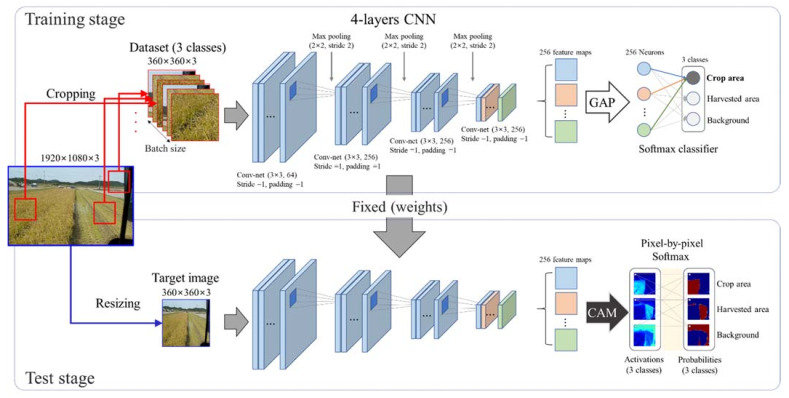
Proposed weakly supervised crop area segmentation. The upper and lower lines show the processes for partial image-based classification model training and full image-based area segmentation testing, respectively.

Figure 2 shows the proposed weakly supervised crop area segmentation (WSCAS) method that consists of two stages: training and testing. The main technique is that partial images cropped in a class-specific area in a raw image are used to train the classification model; then, the target object (crop area) is segmented from the raw image based on implicitly learned localization. In this way, effective segmentation can be achieved even on a small data scale. Four layers of a conv-net (convolutional neural network) consisting of max-pooling and rectified linear unit (ReLU) non-linearity were used as a backbone, after which global average pooling (GAP) was added to generate CAMs to represent the localization of the class-specific image area.

In the training stage, class-specific images cropped from a raw image were used as inputs for the classification model; the number of final feature maps was 256 and each map had a 1/8 input size (width × height). The 256 feature maps were represented into 256 neurons by GAP, which averages each feature map and forms it into a score (neuron), as shown in Equation (1). Then, the neurons were fully connected to output neurons for class-specific scoring. The weights between these connections indicated the importance of each final feature map for each class and contained implicit localization cues. The final output was represented by the probabilities between the scores for the three classes (crop, harvested area, background) by the softmax classifier.

In the test stage, the model weights were fixed, and a resized raw image equal in size to the cropped image used for training was the input of the model. Extracted feature maps from the final conv-net layer were used to generate the CAMs that were calculated by summing the feature maps multiplied by their weight to correspond to the classes, as shown in Equation (2). The visualized CAMs show the pixel-level activations related to the corresponding classes, which can be used as the localization results. Finally, categorical softmax, as shown in Equation (3), was conducted in a channel-based manner pixel by pixel, and spatially identical pixels in each CAM had a probability distribution for its class. The target class can be segmented by pixel-level classification.

(1) $$G_{k}=\frac{\sum_{x,y}f_{k}\left(x,\;y\right)}{N}$$

Here, *G_k_* is the global average pooling of the *k*th feature map, *f_k_(x, y)* is the pixel value at the *x*th row, *y*th column in the *k*th feature map, and *N* is total number of pixels in the feature map.

(2) $$M_{c}\left(x,\;y\right)=\sum_{k}\omega_{k}^{c}\cdot f_{k}\left(x,\;y\right),$$

Here, *M_c_*(*x*, *y*) is the activation value at pixel (*x*, *y*) for the *c*th class and *ω_k_^c^* is the weight of the *k*th neuron from GAP for the *c*th class.

(3) $$P_{c}\left(x,\;y\right)=\frac{exp\left(M_{c}\left(x,\;y\right)\right)}{\sum_{c}exp\left(M_{c}\left(x,\;y\right)\right)}$$

Here, *P_c_*(*x*, *y*) is the probability at pixel (*x*, *y*) for the *c*th class.

### 2.3. Experiments

Field experiments were carried out at a rice paddy field in Korea (35°32′20.2″ N, 128°28′14.7″ E) on 0.14 ha (1400 m^2^ = 70 m × 20 m) plots. The weather was clear and sunny during the tests. An 85 ps combine harvester (DXM85GF, Daedong Co., Ltd., Daegu, Korea) was used to collect field image data and a webcam (C922 Pro, Logitech, Fremont, CA, USA) with a single-board PC (LattePanda Alpha, LattePanda, Shanghai, China) was equipped inside the cabin of the combine harvester. The front scenes were recorded at 30 fps and each frame was stored with a resolution of 1920 × 1080 pixels. The test was repeated five times and each test was conducted during forward direction harvesting. The total recording time was approximately 5 min, and a frame was sampled every 15 frames (2 Hz); thus, the total number of acquired images (frames) was 600, and 480 and 120 samples were used for the model training and testing, respectively.

For the model training, each image was cropped in a class-specific manner for three classes. As a result, the training set was composed 1440 samples, including 480 samples per class. Half of the cropped samples were used to train the model, while the other half were used for validation. The samples were inputted into the CNN model by random cropping, with 360 × 360 pixels, which was the input size of the model, used for each iteration. The input size was determined experimentally to have the smallest size that can extract features for crop area classification [24]. Mean squared error (MSE) loss was used to calculate gradients, and the model weights were updated using an Adam optimizer with a learning rate 1 × 10^−4^. The CNN model was repeatedly trained with a batch size of 64, and an early termination method was used to halt the process at the lowest validation cost to prevent overfitting.

The model was tested for crop area segmentation using 120 test images that were resized to 360 × 360 pixels and input into the model. The CAM for the uncut crop area was used to segment the area. The connected component labelling method was used to cluster the pixels within a neighborhood. This method can be used to eliminate small areas that cluster due to a classification error [28]. The model training and testing were implemented in Python 3.7 with PyTorch 1.1; the CPU and GPU used for the image training were an i7-8700K and NVIDIA Titan-V, respectively.

### 2.4. Uncut Crop Edge Detection

**Figure 3:**
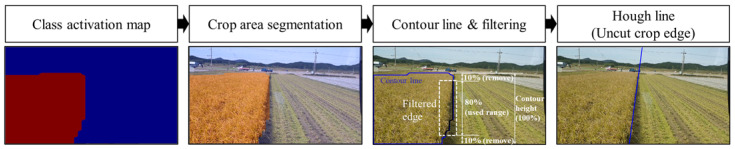
Image processing for determining the uncut crop edge based on segmentation results.

The uncut crop edge for the path guidance of combine harvesters was detected based on the segmentation results, as shown in Figure 3. The uncut crop edge is the boundary in contact with the harvested area and a representative straight line was drawn by Hough transformation [29] based on the contour line. The contour line was detected by the Canny edge detector [30], which is a popular multi-level edge-detection algorithm. The contour lines of the segmented area have four side boundaries: left, right, top, and bottom. The right side is related to the uncut crop edge. Thus, the rightmost pixels were filtered after removing 10% from the top and bottom of the contour area, which made it easy to leave only the right edge of the target area in the scope of this study. The Hough transformation can generate lines based on pixels of filtered contour lines. These were averaged and used as a single line for the final determination of the uncut crop edge.

### 2.5. Performance Evaluation

The proposed WSCAS was evaluated by comparing it with previous deep-learning-based segmentation methods: FCN [16] and image-patch classification (IPC)-based area segmentation [24]. FCN is a well-known model for object semantic segmentation, and the VGG16-FCN8s architecture was used in this study. The IPC has in recent years been proposed as an efficient segmentation method for agricultural navigation. The patch size in this study was set to 80 × 80 pixels, the minimum size for extracting crop-area features. Criteria were selected for two perspectives: area segmentation and line detection. The former was evaluated using the intersection over union (IoU) and inference time, while the latter was evaluated on lateral and angular errors to the target line [24]. The ratio of the distance between the lowest points of the estimated and target lines and the width of the image was used as the lateral error, while the difference in angle between the two lines was used as the angular error. The ground truth of the crop area was annotated manually to calculate the IoU as a polygon, and the target edge was the right boundary (straight line) of the ground truth.

To compare the performances among the three methods statistically, one-way analysis of variance and least significant difference tests were conducted with SAS (version 9.1, SAS Institute, Cary, NC, USA) using the segmentation method as a factor.

## 3. Results

### 3.1. Crop Area Segmentation

**Figure 4:**
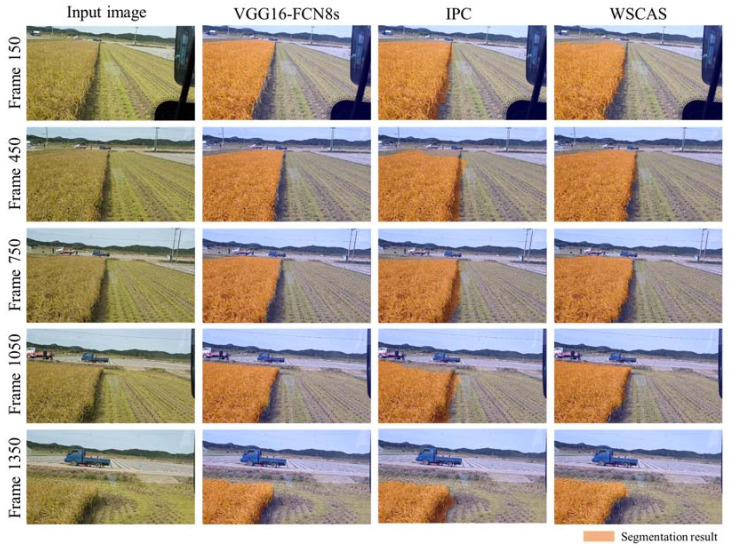
Representative segmentation results for several frames that have 300 regular intervals from the top to the bottom row. Each row consists of 4 images: the input image and the segmentation results using VGG16-FCN8s, IPC, and WSCAS from left to right.

Figure 4 shows the representative segmentation results achieved by each method for five example images. Each row depicts the input image as well as the segmentations via the FCN, IPC, and proposed WSCAS. The frames were sampled with 300 intervals from the frame 150 to 1350. The higher the frame number, the longer the working time, and the more the crop area in the FOV decreased. After frame 1500, the crop area (both uncut and harvested) did not exist or was mostly gone and was not displayed in the results. The results by three methods mostly depicted satisfactory performances because the crop area was segmented correctly; however, there was a performance difference at the boundary according to each method. While the FCN segmented the crop area with clear upper and right boundaries, the other segmentation methods had a low boundary resolution. In particular, the IPC had a step shape at the right boundary and included large amounts of the harvested area compared to other methods. This was because the IPC segmented the target regions with at the image patch level rather than pixel level, and the performance depends on the patch size. It also could not segment the upper crop area for this reason. The WSCAS showed a slightly lower performance than that of the FCN; however, it was observed to show the significant performance generally compared with the FCN, considering that the WSCAS was conducted based on image-level annotation rather than pixel-level annotation.

**Figure 5:**
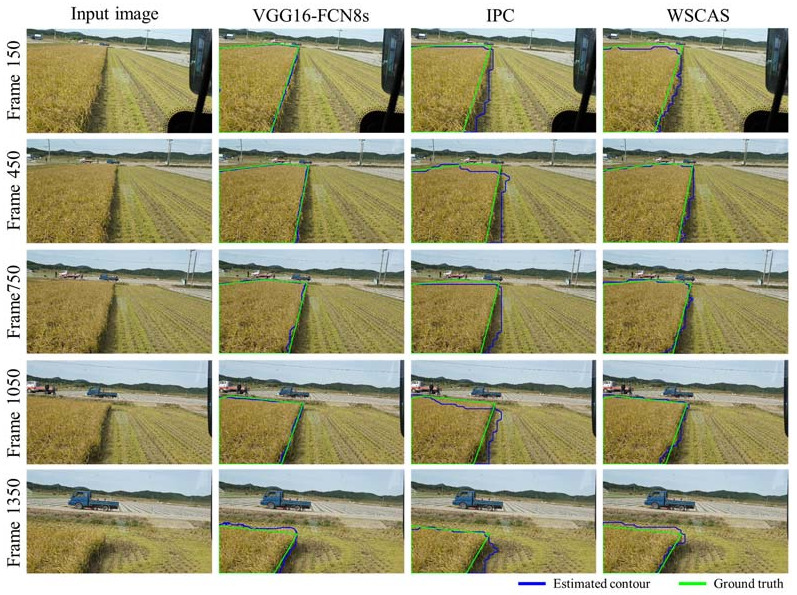
Representative contour lines for several frames that have 300 regular intervals from the top to the bottom row. Each row consists of four images: the input image and the segmentation results using VGG16-FCN8s, IPC, and WSCAS from left to right.

These results can be observed better in Figure 5, which shows the contour lines for the segmented results compared with ground truth. The FCN that trained the model for pixel-level classification showed a robust segmentation independent of distortion from the perspective effect of the 2D scene. The IPC did not represent a diagonal line at the target edge on the right side, and the edge consisted mostly of vertical lines due to the patch-level classification, where the patch size was 80 × 80 pixels. The WSCAS performed better than the IPC although the linearity of the boundary was lower than that of the FCN.

**Figure 6:**
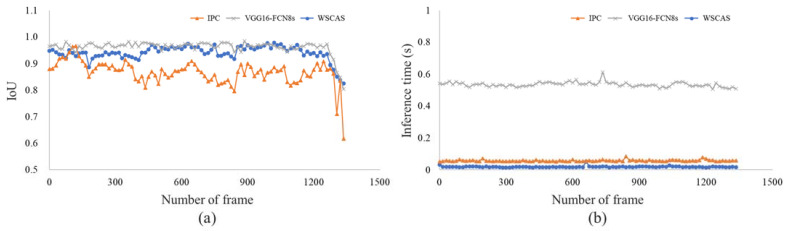
Localization performance (**a**) and inference time (**b**) comparisons by the segmentation methods.

Figure 6 shows the localization performance and inference time by the segmentation methods for test frames. Localization was evaluated by the IoU metric; in most frames, the IoU had the highest performance with the FCN. Our approach, WSCAS, performed comparably with FCN in terms of localization and had over 0.9 IoU from frame 0 to 1200, after which localization performance was lower in all methods. It might be that the error ratio to target area was relatively increased due to a decrease in target area. In addition, the target area was composed of closely located crops that were observed in detail, but the detailed crop shape, including stem, prevented linear boundary detection.

Inference time means the time spent segmenting the target area. WSCAS showed the fastest time of <0.1 s, as did the IPC. Considering only the inference time, FCN may appear to be slower than the other methods in terms of processing speed. However, it is difficult to say that the processing speed of the FCN is poor compared to the others because it has deeper networks and more parameters; CNN has just four layers. It can be inferred that it is more effective to optimize performance in a limited range (segmenting just a few objects) by a using smaller dataset and a shallow model rather than laboriously constructing a large-size labeled dataset to find deep features. For these reasons, WSCAS has a strong advantage in agriculture navigation, such as the path guidance of the combine harvester investigated in this study.

### 3.2. Uncut Crop Edge Detection

**Figure 7:**
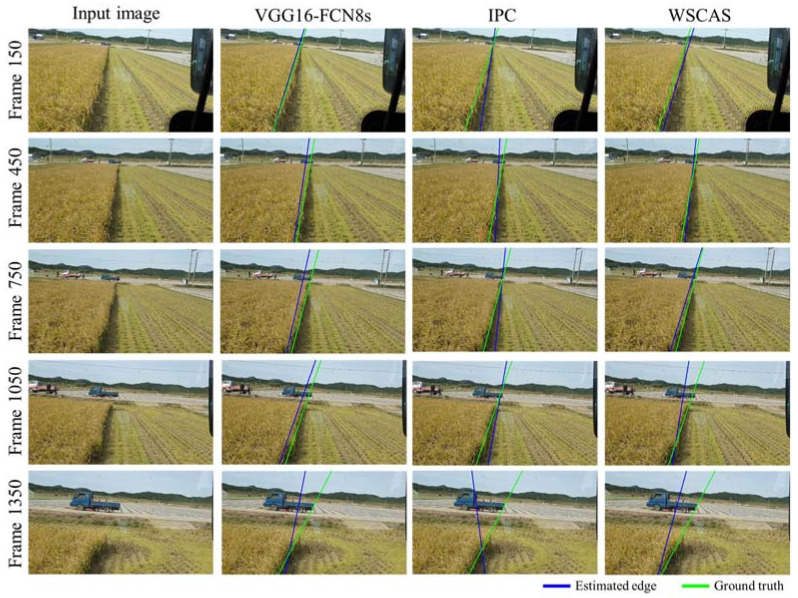
Representative results of edge detection for several frames that have 300 regular intervals from the top to the bottom row. Each row consists of 4 images: the input image and the segmentation results via VGG16-FCN8s, IPC, and WSCAS from left to right.

Figure 7 shows the estimated edge of the uncut crop area in comparison with ground truth. The uncut crop edges can be well detected near the actual edges in the three methods; the results by the IPC were relatively poor compared to those of the other methods. At the beginning and middle of harvesting (frames 150, 450, and 750), the FCN- and WSCAS-based edge detections showed paths nearly identical to the estimated path and ground truth although there were some offset and angle differences. At the end of the work (frame 1350), a significant angular error increase was observed in all methods, which may be due to the uneven contour lines caused by crop interference as mentioned above. The determined edge was represented as a straight line by averaging the value of the possible Hough lines. The results vary depending on how one determines the representative line for the uncut crop edge. This is an important issue for the path guidance of a combine harvester. However, we focused on segmentation suited to agricultural navigation using deep learning, and our proposed method showed a comparable performance to those of the general semantic segmentation methods.

**Figure 8:**
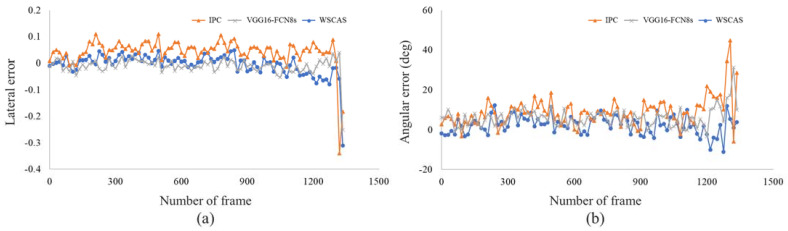
Lateral (**a**) and angular (**b**) errors of the estimated uncut crop edge for path guidance.

The lateral and angular errors of the segmentation methods were represented by frames, as shown in Figure 8. The interval of each point on the graph is 15 frames, which was used as the sample rate. From the beginning to frame 1200, the lateral error ranged from −0.05 to 0.05 for the FCN and WSCAS, while the IPC showed an approximate range of 0–0.1 in the positive direction. In the case of the IPC, the estimated edge was shifted from the actual one because some harvested area was in segmented area. After frame 1200, the lateral error gradually increased in the range of −0.1 to 0.1 then sharply dropped after around frame 1300 because the vertical contour lines for determining the edge were significantly reduced making it difficult to determine the linear direction of the edge. The angular error fluctuated in the range of 0–20° from frame 1200, while the error range increased thereafter similarly to the lateral error. In most of frames, the absolute value of the angular error was similar in WSCAS and the FCN and highest in the IPC.

### 3.3. Comparisons

Table 1 shows a performance comparison according to the segmentation methods using statistical analysis. The IoU as a localization metric was highest in the FCN model with a value of 0.96. WSCAS localized the crop area, with an IoU of 0.94, which is similar to that of the FCN. The FCN used the deeper layer to train the pixel-level segmentation, and it is possible that it showed the highest performance; however, it required the longest inference time (0.54 s) for segmentation due to the large number of parameters that needed to be trained. The WSCAS and IPC-based segmentations were processed more quickly than the FCN; in fact, WSCAS not only segmented the crop area with a high performance but was also three times faster than the IPC and 27 times faster than the FCN. This result cannot be generalized over all semantic segmentations, but it is at least significant for area detection in agricultural navigation. The proposed method ensures fast performance and avoids the laborious large-scale data collection associated with pixel-level annotations.

The IPC the highest value for lateral and angular errors on the determined edge, whereas the lateral error for the WSCAS was acceptable considering the FCN error, while the angular error for the WSCAS was the lowest among the three methods.

## 4. Discussion

In this study, crop area segmentation was proposed based on weakly supervised learning. The results showed that the proposed method not only achieved localization comparable to established deep-learning-based semantic segmentation methods, but also reduced the inference time for crop area detection. The proposed method, WSCAS, operated faster than the FCN8s and had similar localization performance. The proposed segmentation had an IoU of 0.94, and the inference time was approximately 0.02 s, a 96.3% reduction compared to the FCN8s. For semantic segmentation, image patch-based classification required weaker supervision than pixel-based classification and can be configured with shallow CNN layers. For this reason, IPC-based segmentation showed fast inferences in real-time despite a decrease in localization performance. Nevertheless, WSCAS segmented the crop area 3 times faster with 8% higher localization than IPC-based segmentation. Our method also had outstanding performances in recent studies on semantic segmentation for robotic harvesters (IoU = 0.9, inference time = 0.031 s) [14] and orchard path detection (IoU = 0.75, inference time = 0.11 s) [24].

Regarding uncut crop edge detection, this study showed that the proposed method detected the edge with 0.026 and 4.22° errors for lateral and angular deviations, respectively, and were similar error range to path detection in agricultural navigation: orchard path detection (lateral error = 0.02–0.1, angular error = 2.6–3.3°) [24], tillage boundary detection (angular error = 8.9°) [31], and tractor path-tracking simulation(angular error = −8–5°) [32].

These results showed that our WSCAS method, including object-level localization, used weakly supervised learning to infer an object in a monotonous scene like a frontal image during combine harvesting effectively. The general deep-learning-based semantic segmentation (e.g., FCN) required a large number of parameters (deep network) to segment the various objects accurately and robustly; however, agricultural navigation has few objects with little movement, so even a shallow model is sufficient to extract features that can discriminate essential classes. Although deep networks may have good scalability for various agriculture environments, it is impossible to collect data that can include agricultural diversity; thus, applying a fast and simple learning process to the target domain was effective. Our method trained the model to use a small amount of data captured immediately from the field to infer the target region from a frontal FOV by training just class-specific images with image-level labeling.

The results showed that WSCAS can perform comparable to previous segmentation, and it is possible to use as real-time application. However, despite these advantages, there are limitations that need to be addressed. The coverage of generalization was not tested under the environmental conditions such as weather, land type, slope, and illumination, but the issue such as illuminance change within one domain can tackle by data augmentation and fine-tuning. Nevertheless, domain diversity in agriculture by various factors like crop, soil, field shape, work type is not easy to be covered by our method without increasing model parameters. It is expected that this will be solved by a long-term study to devise a strategy based on model optimization and target-site clustering.

## 5. Conclusions

In this study, WSCAS was proposed to overcome the problem of a lack of resources for deep learning in agricultural navigation. It determined the uncut edge of by effective and rapid crop-area segmentation in real time. WSCAS learned the localization indirectly using image-level labelling, and the model was configured shallowly, which is suitable for monotonous front-facing scenes of agricultural navigation. It showed advantage of usability for crop area segmentation by reducing the burden of data construction and model training.

Comprehensively, it can be seen that this study derived meaningful results for machine-vision-based navigation in agriculture since it was possible to infer the target from the entire image by learning fragmentary information from a small-scale data set that can be constructed easily and facilitates model implementation. The proposed method can be used in field conditions by optimizing the model parameters, evaluating real performance based on location measurement, and designing the line detection and homography to find the real coordinates of the uncut edge.

## Figures and Tables

**Table 1:** Statistical comparison of the performances by the segmentation methods.

Method	Base Model	IoU	Inference Time (s)	Lateral Error	Angular Error (°)
FCN8s	VGG16	0.96 ± 0.026 ^a^	0.54 ± 0.015 ^a^	0.019 ± 0.027 ^b^	6.23 ± 4.605 ^b^
IPC	4-layer CNN	0.87 ± 0.045 ^c^	0.06 ± 0.005 ^b^	0.055 ± 0.041 ^a^	9.39 ± 6.968 ^a^
WSCAS	4-layer CNN	0.94 ± 0.027 ^b^	0.02 ± 0.004 ^c^	0.026 ± 0.035 ^b^	4.22 ± 2.964 ^c^

Average ± standard deviation. Means with different superscripts (a,b,c) in each column are significantly different at *p* < 0.05 by the LSD multiple-range test.

## Data Availability

Not applicable.
